# Explaining Order among Those Who Share Positions in the Author Byline

**DOI:** 10.1128/mBio.01981-19

**Published:** 2019-09-17

**Authors:** Arturo Casadevall, Patrick Schloss

**Affiliations:** a Johns Hopkins Bloomberg School of Public Health, Baltimore, Maryland, USA; b Microbiology & Immunology, University of Michigan—Ann Arbor, Ann Arbor, Michigan, USA

**Keywords:** authorship, bias, gender

## Abstract

In an effort to reduce gender biases in authorship positions, *mBio* will now require an explanation for how order was determined among those who share positions in the author byline. We hope that by requiring explanations, the explanation will help those in second and third positions get their full share of credit, even when not listed first.

## EDITORIAL

Two studies have shown gender-related inequities in the ordering of authors sharing the first position in biomedical research articles, with males being favored (1, 2). An analysis by the *Journal of Clinical Investigation* into recent publications also revealed gender inequities in author order, with males being favored in the first position (3). Although the mechanism by which these inequities occur is uncertain, there is concern that conscious and unconscious biases may contribute to their emergence. This issue is important because review and promotion committees may not give equal credit to first and second authors, even when they are stated to have contributed equally, and bias against females in author order determination may be detrimental to their scientific careers. Given the evidence from these studies, the *Journal of Clinical Investigation* instituted the requirement that authors explain how ordering was done when two or more individuals share the first author position (3). The rationale for this decision was to give the authors the opportunity to state how these decisions were made, which may help those in the second and third positions explain why they were listed there despite sharing the first author position. Furthermore, such a requirement would necessarily lead to discussions between authors, and we believe that such discussions may lead to fairer decisions.

At the June 2019 meeting of the editors in chief of the American Society for Microbiology journals in San Francisco, CA, the editors discussed the issues of author order and gender and voted unanimously to adopt the requirement that the rationale for author order be stated in the manuscript. *mBio* will now require that authors provide an explanation for how author order was determined in situations where two or more individuals share the first position as a footnote to the title. The journal does not mandate, favor, or champion any particular method for determining how author order is determined for those sharing the first position but does want an explanation. We are hopeful that this step will help authors receive their full share of the credit. For example, such explanations may be included in curriculum vitae in which the co-first author is in a position other than first in the byline. Although we recognize that this is a small step in redressing the problem of inequities and biases, we are hopeful that there will be circumstances when these explanations will be helpful to some authors in obtaining their full share of credit and hope that other journals adopt similar policies.

## References

[1] Broderick NA, Casadevall A 2019 Gender inequalities among authors who contributed equally. Elife 8:e36399. doi:10.7554/eLife.36399. 30698140 PMC6353592

[2] Aakhus E, Mitra N, Lautenbach E, Joffe S 2018 Gender and byline placement of co-first authors in clinical and basic science journals with high impact factors. JAMA 319:610–611. doi:10.1001/jama.2017.18672. 29450515 PMC5838607

[3] Casadevall A, Semenza GL, Jackson S, Tomaselli G, Ahima RS 2019 Reducing bias: accounting for the order of co-first authors. J Clin Invest 130:2167–2168. doi:10.1172/JCI128764. PMC6546447 31033485

